# Association between spicy food and hypertension among Han Chinese aged 30–79 years in Sichuan Basin: a population-based cross-sectional study

**DOI:** 10.1186/s12889-023-16588-6

**Published:** 2023-08-30

**Authors:** Liling Chen, Rui Ding, Qinwen Luo, Xiaojun Tang, Xianbin Ding, Xianxian Yang, Xiang Liu, Zhifeng Li, Jingru Xu, Jiantong Meng, Xufang Gao, Wenge Tang, Jing Wu

**Affiliations:** 1 Institute of Chronic Non-Communicable Disease Control and Prevention, Chongqing Center for Disease Control and Prevention, Chongqing, 400042 China; 2 National Center for Chronic and Noncommunicable Disease Control and Prevention, Chinese Center for Disease Control and Prevention, Beijing, 100050 China; 3 First Medical College, Chongqing Medical University, Chongqing, 400016 China; 4 School of Public Health and Management, Research Center for Medical and Social Development, Chongqing Medical University, Chongqing, 400016 China; 5 Department of Epidemiology and Health Statistics, West China School of Public Health, Sichuan University, Chengdu, 610041 China; 6 Chengdu Center for Disease Control and Prevention, Chengdu, 610047 China

**Keywords:** Spicy food consumption, Hypertension, Blood pressure, Cross-sectional study, Sichuan Basin

## Abstract

**Background:**

While spicy food is believed to have cardiovascular-protective effects, its impact on hypertension remains uncertain due to conflicting findings from previous studies. This study aimed to explore the association between spicy food and hypertension in Sichuan Basin, China.

**Methods:**

The baseline data of 43,657 residents aged 30–79 in the Sichuan Basin were analyzed including a questionnaire survey (e.g., sociodemographics, diet and lifestyle, medical history), medical examinations (e.g., height, body weight, and blood pressure), and clinical laboratory tests (e.g., blood and urine specimens). Participants were recruited by multi-stage, stratified cluster sampling in consideration of both sex ratio and age ratio between June 2018 and February 2019. Multivariable logistic regression was performed to explore the effect of spicy food on hypertension and multivariable linear regression was applied to estimate the effect of spicy food on systolic and diastolic blood pressure (SBP/DBP).

**Results:**

Concerning hypertension, negative associations with spicy food consumption were observed only in females: compared to those who do not eat spicy food, the odds ratios of consuming spicy food 6–7 days/week, consuming spicy food with strong strength, and years of eating spicy food-to-age ratio were 0.886 (0.799, 0.982), 0.757 (0.587, 0.977), 0.632 (0.505, 0.792), respectively. No significant association was found in males (All *P* trends > 0.05). In the stratified analyses, participants in the subgroup who were 50 to 79 years old (OR, 95%CI: 0.814, 0.763, 0.869), habitually snored (OR, 95%CI: 0.899, 0.829, 0.976), had a BMI < 24 kg/m^2^ (OR, 95%CI: 0.886, 0.810, 0.969), had a normal waist circumference (OR, 95%CI: 0.898, 0.810, 0.997), and had no dyslipidemia (OR, 95%CI: 0.897, 0.835, 0.964) showed a significantly stronger association. For SBP, consuming spicy food had negative effects in both genders, but the effect was smaller in males compared to females: among males, the β coefficients for consuming spicy food 1–2 days/week, weak strength, and years of eating spicy food-to-age ratio were 0.931 (-1.832, -0.030), -0.639 (-1.247, -0.032), and − 2.952 (-4.413, -1.492), respectively; among females, the β coefficients for consuming spicy food 3–5 days/week, 6–7 days/week, weak strength, moderate strength, and years of eating spicy food-to-age ratio were − 1.251 (-2.115, -0.388), -1.215 (-1.897, -0.534), -0.788 (-1.313, -0.263), -1.807 (-2.542, -1.072), and − 5.853 (-7.195, -4.512), respectively. For DBP, only a positive association between the years of eating spicy food-to-age ratio and DBP was found in males with β coefficient (95%CI ) of 1.300 (0.338, 2.263); Little association was found in females (all *P* trends > 0.05), except for a decrease of 0.591 mmHg ( 95%CI: -1.078, -0.105) in DBP among participants who consumed spicy food 1–2 days/week, compared to those who did not consume spicy food.

**Conclusion:**

Spicy food may lower SBP and has an antihypertensive effect, particularly beneficial for women and individuals with fewer risk factors in the Sichuan Basin. Spicy food consumption may decrease DBP in women but increase it in men. Further multicenter prospective cohort studies are needed to confirm these findings.

**Supplementary Information:**

The online version contains supplementary material available at 10.1186/s12889-023-16588-6.

## Introduction

Hypertension is a major health problem and one of the most prevalent as well as modifiable risk factors for cardiovascular diseases (CVDs) [1, 2]. High systolic blood pressure (SBP) has been recognized as the leading cause of attributable deaths around the world and was estimated to account for 19.2% of total deaths (10.8 million deaths) in 2019 [3]. A nationally representative survey indicated that about 244.5 million (23.2%) Chinese adults had hypertension, and about 435.3 million (41.3%) had prehypertension [4]. Indeed, there is growing evidence that dietary modification plays a critical role in the prevention and treatment of hypertension [5].

Spices have a long history of being used for flavoring, coloring, and preserving food, even for medicinal purposes, but overall, the health effects of eating spicy food remain uncertain [6]. Some studies demonstrated that consuming spicy food had a beneficial influence on total and specific mortality [7, 8], lipid profiles [9, 10], as well as cancer [11–13]. While others suggested that spicy food consumption may have adverse or no effect on diseases such as dyslipidemia [14, 15], diabetes mellitus [16], obesity [17–19], hyperuricemia [20, 21], and cancer [22]. Regarding the association between spicy food and hypertension, results from previous studies are also conflicting. Meta-analysis of clinical trials [23] showed no significant effect of red pepper/capsaicin on blood pressure (BP). Conversely, observational studies [24–26] demonstrated that spicy food is inversely associated with hypertension among adults. In addition, Harada et al. [27] illustrated that SBP and diastolic blood pressure (DBP) were significantly lower among hypertensive volunteers after the administration of a mixture of capsaicin and isoflavone for 5 months.

Spicy food consumption habits and their relationship with lifestyle behaviors vary across regions [28, 29], which may contribute to inconsistent findings compared to previous research. Exploring the associations between spicy food and hypertension in different regions and among individuals with diverse dietary habits holds significant practical implications. Sichuan Basin is located in southwest China, where the climate is humid and residents prefer spicy flavors [18]. Therefore, this study aimed to explore the effect of spicy food consumption on hypertension based on the baseline data from the China Multi-Ethnic cohort (CMEC) study in the Sichuan Basin.

## Materials and methods

### Study Population

We gathered the baseline data from the CMEC in Sichuan Basin (Sichuan and Chongqing region) from June 2018 to February 2019, including questionnaire survey (e.g., sociodemographics, diet and lifestyle, medical history), medical examinations (e.g., height, body weight, and blood pressure), and clinical laboratory tests (e.g., blood and urine specimens). A total of 44,900 Han ethnicity permanent residents aged 30–79 were recruited by multi-stage, stratified cluster sampling in consideration of both sex ratio and age ratio between June 2018 and February 2019. More details about the study design have been described previously [18, 30]. After excluding those with missed data on hypertension (*n* = 698), frequency of spicy food (*n* = 38), or other confounding factors (*n* = 507, such as BMI, physical activity, and DASH score), 43,657 participants were included in the final analysis. The informed consent was signed by all subjects involved in the study. This study was approved by the Sichuan University Medical Ethical Review Board (K2016038), the Research Ethics Committee of Chongqing Center for Disease Control and Prevention (2017(001), 2021(006)).

### Outcome variables

Blood pressure of the right arm was measured by trained medical staff using electronic sphygmomanometers at an interval of one minute in the resting condition three times consecutively. To minimize the impact on blood pressure measurement values, participants were informed in advance not to smoke, drink alcohol, consume coffee or tea, or engage in physical exercise before blood pressure measurement. SBP/DBP used in the analysis was the average of three readings. Hypertension was defined as SBP/DBP ≥ 140/90 mmHg or a history of hypertension diagnosed by doctors [31, 32].

### Assessment of spicy food consumption

We used a validated dietary habit questionnaire to collect information about the frequency and strength of spicy food consumption as well as the age they started to eat spicy food. Frequency of spicy food consumption was estimated through the question “How often did you consume spicy food during the past month? ”, with the following five response categories: “No”, “<1 day/week”, “1–2 days/week”, “3–5 days/week” and “6–7 days/week”. “No” means no consumption of spicy food; “<1 day/week” means consuming spicy food less than once per week; “1–2 days/week” means consuming spicy food 1–2 days per week; “3–5 days/week” means consuming spicy food 3–5 days per week; and “6–7 days/week” means consuming spicy food 6–7 days per week. Participants who ate spicy food at least one day per week were further asked “What strength of spicy food do you usually eat?”, with three response categories: “weak”, “moderate”, and “strong”. Furthermore, they were also asked, “What age did you start to eat spicy food ?” If the respondents have had the habit of consuming spicy food weekly since childhood, we uniformly considered the age as 5 years old. Hence, years of eating spicy food to age ratio were calculated as another measurement.

### Assessment of covariates

A face-to-face electronic questionnaire survey was performed by well-trained interviewers who were typically local college students with medical backgrounds to collect sociodemographic characteristics and lifestyle factors. Smoking status was categorized into three groups: “never”, “former”, and “current”, respectively representing respondents who have never smoked, respondents who used to smoke but have quit, and respondents who currently smoke at the time of the survey. Physical activity was converted into the metabolic equivalent task of hours per day (METs-hours/day) spent on occupation, transportation, housework, and leisure time activities [33]. Snoring was self-reported with “no”, “occasionally” and “habitual”. Family history of hypertension refers to self-reported hypertension from at least one first-degree relative (biological parents, siblings).

A food frequency questionnaire (FFQ) including 13 major food groups according to the Chinese Dietary Guidelines and the eating habits of southwestern Chinese was delivered to each participant to collect their dietary intake information [34]. Consumptions of alcohol, tea, cooking oil, and salt were also collected in separate sections. Alcohol drinking was calculated as grams of pure alcohol per week, based on the self-reported alcohol type, amount drunk, and frequency, assuming the following alcohol content by volume (v/v) in China: beer 4%, grape wine 12%, rice wine 15%, weak spirits 38% and strong spirits 53% [35]. Total daily energy intake was estimated according to the 2018 China food composition tables and the China food exchange lists (standard edition) [36, 37]. The Dietary Approaches to Stop Hypertension (DASH) score was obtained by summing up the scores of seven kinds of food including whole grains, fresh fruits, vegetables, beans, red meat products, dairy, and sodium salt, ranging from 7 (minimal adherence) to 35 (maximal adherence). We conducted repeated FFQ and 24-hour dietary recall (24 HDRs) to assess the reproducibility and validity of the baseline FFQ. Regarding reproducibility, intraclass correlation coefficients (ICC) for food groups ranged from 0.15 for fresh vegetables to 0.67 for alcohol. Regarding validity, de-attenuated Spearman rank correlation coefficients for food groups ranged from 0.10 for soybean products to 0.66 for rice. More details are provided in our previous study [34].

Body mass index (BMI) was calculated using weight divided by height squared (kg/m^2^). Waist circumference (WC) was measured using a non-stretchable soft tape at the midpoint between the lowest rib and the iliac crest, with an accuracy of 0.1 cm. Blood samples after fasting for 12 h were collected to test the lipid profiles, fasting blood glucose, and glycosylated hemoglobin percentage. Dyslipidemia was regarded as having any one of the following conditions: (1) triacylglycerol (TG) ≥ 2.26 mmol/l; (2) serum total cholesterol (TC) ≥ 6.22 mmol/l; (3) low-density lipoprotein cholesterol (LDLC) ≥ 4.14 mmol/l; (4) and high-density lipoprotein cholesterol (HDLC) < 1.04 mmol/l; (5) a history of hyperlipemia diagnosed by a physician [38]. Diabetes was diagnosed as fasting blood glucose (FBG) ≥ 7.0 mmol/L, a glycosylated hemoglobin percentage of ≥ 6.5%, or a self-reported diagnosis of diabetes by a physician [39].

### Statistical analysis

Descriptive statistics were presented as numbers (percentages) or medians (Q1, Q3) for categorical or continuous variables, respectively. The chi-square test and Kruskal-Wallis H test were performed to compare the differences in covariates among various groups of spicy food intake. Multiple logistic regression analyses were performed to estimate the odds ratios (OR) and corresponding 95% confidence interval (CI) of spicy food consumption with hypertension. Multiple linear regression models were applied to explore the coefficients (95% CI) for SBP/DBP. Potential confounding factors were progressively adjusted in a series of models. Model 1 was the crude model without any adjustments; Model 2 adjusted for age, gender, household income (< 12,000 yuan, 12,000–19,999 yuan, 20,000–59,999 yuan, 60,000–99,999 yuan, ≥ 100,000 yuan), and family history of hypertension (yes or no); Model 3 adjusted for model 2 plus smoking status (never, former and current), alcohol drinking (continuous), physical activity (continuous), total energy intake per day (continuous), DASH score (continuous), snoring (no, occasionally, habitual); Model 4 adjusted for model 3 plus BMI (continuous), WC (continuous), dyslipidemia (yes or no) and diabetes (yes or no). Stratified analyses were conducted to detect whether the associations of spicy food consumption with prevalent hypertension differed according to covariates. Furthermore, sensitivity analyses were performed by excluding participants who reported having peptic ulcer diseases. Statistical analyses were conducted using SPSS version 25.0. The forest plot was drawn by R 4.1.1. All statistical tests were two-sided, and a *P* value < 0.05 was considered statistical significance.

## Results

### General characteristics

Table 1 presents the general characteristics by frequency of spicy food consumption. Of the 43,657 subjects, the median age was 49.75 (42.83, 61.08) years and 54.50% of them were females. 14,519 individuals had hypertension with a prevalence of 33.26%. Participants who consumed spicy food more frequently were more likely to be younger, male, current smokers, and to have a family history of hypertension; to drink more alcohol; to have higher household income, higher total energy intake, higher DASH scores, and higher levels of physical activity; but were less likely to be snorers (all *P* trend < 0.001). Among those who ate spicy food > 1 day/week, participants who ate spicy food more frequently were more likely to eat spicier, eat spicy at an earlier age, eat spicy longer, and have a greater ratio of years of spicy eating to age (all *P* trend < 0.001).

**Table 1:** Baseline characteristics of participants according to the frequency of spicy food consumption

	Overall	Frequency of spicy food consumption					*P* trend
		No	<1 day/week	1-2 days/week	3-5 days/week	6-7 days/week	
No.participants	43657 (100.00)	5116(11.70)	3909(9.00)	6987(16.00)	5242(12.00)	22403(51.30)	
Ages (years)	49.75 (42.83,61.08)	59.42 (48.17,68.08)	52.08 (44.50,63.08)	48.08 (41.33,57.92)	47.08 (39.50,55.42)	49.50 (42.58,59.58)	<0.001
Gender							
Male	19866(45.50)	2240(43.78)	1592(40.73)	2966(42.45)	2389(45.57)	10679(47.67)	<0.001
female	23791(54.50)	2876(56.22)	2317(59.27)	4021(57.55)	2853(54.43)	11724(52.33)	
Household income (Yuan/year)							
< 12000	4444(10.18)	966(18.88)	504(12.89)	647(9.26)	382(7.29)	1945(8.68)	<0.001
12000-19999	5304(12.15)	860(16.81)	556(14.22)	822(11.76)	540(10.30)	2526(11.28)	
20000-59999	15608(35.75)	1890(36.94)	1386(35.46)	2444(34.98)	1837(35.04)	8051(35.94)	
60000-99999	9255(21.20)	806(15.75)	809(20.70)	1547(22.14)	1238(23.62)	4855(21.67)	
≥ 100000	9046(20.72)	594(11.61)	654(16.73)	1527(21.85)	1245(23.75)	5026(22.43)	
Family history of hypertension							
No	26717(61.20)	3511(68.63)	2461(62.96)	4196(60.05)	3048(58.15)	13501(60.26)	<0.001
Yes	16940(38.80)	1605(31.37)	1448(37.04)	2791(39.95)	2194(41.85)	8902(39.74)	
Smoking status							
Never	30844(70.65)	4030(78.77)	3071(78.56)	5253(75.18)	3704(70.66)	14786(66.00)	<0.001
Former	2897(6.64)	342(6.68)	255(6.52)	432(6.18)	324(6.18)	1544(6.89)	
Current	9916(22.71)	744(14.54)	583(14.91)	1302(18.63)	1214(23.16)	6073(27.11)	
Alcohol drinking (grams/week)	5 (0,5)	0 (0,5)	0 (0,5)	5 (0,5)	5 (0,5)	5 (0,5)	<0.001
Total energy intake (kcal/day)	1647.34 (1320.3,2058.05)	1547.97 (1209.23,1935.23)	1561.87 (1241.91,1960.39)	1583.43 (1270.97,1990.09)	1632.47 (1316.81,2019.71)	1711.53 (1377.14,2129.30)	<0.001
DASH score	22(18,25)	22(17,24)	22(18,25)	22(19,25)	22(19, 25)	21(18,24)	<0.001
Physical activity (MET-hours/day)	28.05 (17.63,38.64)	23.54 (14.14,37.07)	26.57 (15.90,38.26)	28.43 (18.16,37.88)	28.64 (19.1,38.4)	28.71 (18.49,39.3)	<0.001
Snoring							
No	10540(24.14)	1111(21.72)	845(21.62)	1351(19.34)	1145(21.84)	6088(27.17)	<0.001
Occasional	11431(26.18)	1030(20.13)	976(24.97)	2027(29.01)	1548(29.53)	5850(26.11)	
Habitual	21686(49.67)	2975(58.15)	2088(53.42)	3609(51.65)	2549(48.63)	10465(46.71)	
BMI	24.28 (22.21,26.56)	24.14 (22.12,26.37)	24.12 (22.14,26.41)	24.01 (22.02,26.26)	24.17 (22.14,26.4)	24.44 (22.31,26.73)	<0.001
Waist circumference	82.5(76.0,89.0)	82.0(76.0,88.1)	81.5(76.0,88.1)	81.0(75.0,88.0)	82.0(75.0,89.0)	83.0(76.2,90.0)	<0.001
Dyslipidemia							
No	29296(67.10)	3423(66.91)	2729(69.81)	4838(69.24)	3531(67.36)	14775(65.95)	<0.001
Yes	14361(32.90)	1693(33.09)	1180(30.19)	2149(30.76)	1711(32.64)	7628(34.05)	
Diabetes							
No	38399(87.96)	4348(84.99)	3409(87.21)	6214(88.94)	4696(89.58)	19732(88.08)	<0.001
Yes	5258(12.04)	768(15.01)	500(12.79)	773(11.06)	546(10.42)	2671(11.92)	
Hypertension							
No	29138(66.74)	2915(56.98)	2528(64.67)	4944(70.76)	3738(71.31)	15013(67.01)	<0.001
Yes	14519(33.26)	2201(43.02)	1381(35.33)	2043(29.24)	1504(28.69)	7390(32.99)	
SBP (mmHg)	125.33 (114.00,139.00)	130.67 (118.33,145.67)	126.33 (114.33,141.00)	123.33 (112.67,136.33)	123.00 (112.33,135.67)	125.00 (113.67,138.67)	<0.001
DBP (mmHg)	78.00 (71.33,85.67)	78.33 (71.67,86.00)	77.67 (71.00,85.33)	77.00 (70.67,84.33)	77.67 (71.00,85.00)	78.33 (71.67,86.00)	<0.001
Strength of spicy food consumption							
No	9025(20.67)	5116(100.00)	3909(100.00)	-	-	-	<0.001
Weak	26457(60.60)	-	-	6165(88.24)	4199(80.1)	16093(71.83)	
Moderate	7274(16.66)	-	-	746(10.68)	977(18.64)	5551(24.78)	
Strong	901(2.06)	-	-	76(1.09)	66(1.26)	759(3.39)	
Age started to eat spicy food	5 (5,15)	-	-	10 (5,20)	8 (5,17)	5 (5,12)	<0.001
Years of eating spicy food	40 (30,49)	-	-	36 (26,46)	36 (27,45)	41 (32,50)	<0.001
Years of eating spicy food-to-age ratio	0.86 (0.70,0.90)	-	-	0.77 (0.59,0.89)	0.83 (0.65,0.89)	0.88 (0.76,0.90)	<0.001

Continuous data were described as the median (Q1, Q3), and statistical significance was assessed by the Kruskal-Wallis H test. Categorical data were summarized as numbers (percentage), and statistical significance was assessed by a chi-square test.

DASH score: the Dietary Approaches to Stop Hypertension (DASH) diet score, MET: metabolic equivalent of task, BMI: body mass index, SBP (mmHg): systolic blood pressure, DBP (mmHg): diastolic blood pressure.

### Association between spicy food consumption and hypertension

After adjusting for confounding factors in model 4, multivariable logistic regression analyses only showed negative associations between spicy food consumption and hypertension in females but not in males (Table 2). Among females, compared with participants who do not eat spicy food, the adjusted ORs (95% CIs) of consuming spicy food for 6–7 days/week, consuming spicy food with strong strength, and years of eating spicy food-to-age ratio were 0.886 (0.799, 0.982), 0.757 (0.587, 0.977), 0.632 (0.505, 0.792), respectively.

**Table 2:** Association of spicy food consumption with hypertension

	*OR* (95%*CI*)				*P* trend for model 4
	Model 1	Model 2	Model 3	Model 4	
Frequency of spicy food consumption					
Total					
No	Ref	Ref	Ref	Ref	**0.055**
<1 day/week	**0.723(0.664,0.788)**	0.978(0.889,1.076)	0.985(0.894,1.085)	0.970(0.879,1.072)	
1-2 days/week	**0.547(0.507,0.590)**	**0.900(0.827,0.979)**	0.924(0.848,1.007)	**0.898(0.822,0.982)**	
3-5 days/week	**0.533(0.491,0.578)**	0.962(0.877,1.054)	0.973(0.886,1.068)	0.922(0.837,1.015)	
6-7 days/week	**0.652(0.613,0.694)**	1.026(0.957,1.101)	0.987(0.919,1.061)	**0.922(0.856,0.992)**	
Males^a^					
No	Ref	Ref	Ref	Ref	0.801
<1 day/week	**0.867(0.762,0.988)**	1.063(0.925,1.222)	1.057(0.918,1.217)	1.048(0.906,1.212)	
1-2 days/week	**0.689(0.616,0.770)**	0.991(0.878,1.119)	0.989(0.874,1.118)	0.935(0.824,1.062)	
3-5 days/week	**0.676(0.601,0.761)**	1.059(0.931,1.203)	1.040(0.912,1.186)	0.969(0.846,1.110)	
6-7 days/week	**0.767(0.700,0.841)**	**1.117(1.011,1.235)**	1.045(0.941,1.159)	0.988(0.887,1.099)	
Females^a^					
No	Ref	Ref	Ref	Ref	**0.042**
<1 day/week	**0.636(0.567,0.714)**	0.935(0.819,1.068)	0.950(0.831,1.087)	0.924(0.805,1.060)	
1-2 days/week	**0.451(0.407,0.501)**	**0.859(0.762,0.969)**	0.899(0.796,1.016)	0.890(0.785,1.008)	
3-5 days/week	**0.420(0.374,0.471)**	0.910(0.796,1.039)	0.941(0.822,1.076)	0.903(0.786,1.037)	
6-7 days/week	**0.547(0.503,0.595)**	0.969(0.878,1.070)	0.958(0.866,1.059)	**0.886(0.799,0.982)**	
Strength of spicy food consumption					
Total					
No	Ref	Ref	Ref	Ref	**0.035**
Weak	**0.695(0.662,0.730)**	0.977(0.924,1.034)	0.970(0.916,1.027)	**0.927(0.874,0.983)**	
Moderate	**0.706(0.661,0.753)**	**1.102(1.024,1.186)**	1.033(0.958,1.115)	0.956(0.884,1.034)	
Strong	**0.862(0.747,0.993)**	0.990(0.847,1.157)	0.885(0.754,1.038)	**0.808(0.686,0.951)**	
Males^a^					
No	Ref	Ref	Ref	Ref	0.829
Weak	**0.768(0.713,0.827)**	1.014(0.935,1.099)	0.987(0.908,1.072)	0.944(0.866,1.028)	
Moderate	**0.807(0.738,0.883)**	**1.197(1.085,1.320)**	1.095(0.989,1.213)	1.006(0.905,1.118)	
Strong	0.948(0.783,1.148)	1.085(0.886,1.329)	0.966(0.784,1.189)	0.892(0.720,1.107)	
Females^a^					
No	Ref	Ref	Ref	Ref	**0.012**
Weak	**0.632(0.591,0.676)**	0.964(0.891,1.042)	0.970(0.896,1.050)	0.929(0.856,1.008)	
Moderate	**0.522(0.473,0.577)**	0.975(0.869,1.094)	0.949(0.844,1.066)	0.899(0.798,1.014)	
Strong	**0.707(0.569,0.880)**	0.939(0.734,1.200)	0.856(0.667,1.099)	**0.757(0.587,0.977)**	
Years of eating spicy food-to-age ratio					
Total	**2.213(1.926,2.543)**	**0.789(0.680,0.915)**	**0.752(0.647,0.875)**	**0.719(0.616,0.840)**	**<0.001**
Males^a^	**1.965(1.620,2.384)**	0.862(0.702,1.057)	**0.820(0.666,1.010)**	0.833(0.671,1.033)	0.097
Females^a^	**2.696(2.195,3.310)**	**0.727(0.585,0.904)**	**0.688(0.552,0.857)**	**0.632(0.505,0.792)**	**<0.001**

Model 1: original model without any adjustments

Model 2: adjusted for age, gender (male or female), household income (< 12 000 yuan, 12 000–19 999 yuan, 20 000–59 999 yuan,60 000–99 999 yuan, ≥ 100 000 yuan), family history of hypertension (yes or no)

Model 3: adjusted for model 2 plus smoking status (never, former, and current), alcohol drinking (continuous), physical activity (continuous), total energy intake per day (continuous), DASH score (continuous), snoring (no, occasionally, habitual)

Model 4: adjusted for model 3 plus BMI (continuous), waist circumference (continuous), dyslipidemia (yes or no), diabetes (yes or no)

^a^without adjustment for gender

### Association between spicy food consumption and SBP

Multivariable linear regression analyses showed that negative associations between spicy food consumption and SBP were both found in males and females, but the effect was smaller in males than in females (Table 3). Among males, compared to the reference group, the β coefficients (95% CIs) of consuming spicy food for 1–2 days/week, consuming spicy food with weak strength, and years of eating spicy food-to-age ratio were − 0.931 (-1.832, -0.030), -0.639 (-1.247, -0.032), and − 2.952 (-4.413, -1.492), respectively. Among females, the β coefficients (95% CIs) of 3–5 days/week, 6–7 days/week, weak strength, moderate strength, and years of eating spicy food-to-age ratio were − 1.251 (-2.115, -0.388), -1.215 (-1.897, -0.534), -0.788 (-1.313, -0.263), -1.807 (-2.542, -1.072), and − 5.853 (-7.195, -4.512), respectively.

**Table 3:** Association between spicy food consumption and SBP

	*β* coefficients (95%*CI*)				*P* trend for model 4
	Model 1	Model 2	Model 3	Model 4	
Frequency of spicy food consumption					
Total					
No	Ref	Ref	Ref	Ref	**<0.001**
<1 day/week	**-3.934(-4.733,-3.135)**	-0.513(-1.222,0.197)	-0.240(-0.943,0.463)	-0.365(-1.044,0.314)	
1-2 days/week	**-6.938(-7.629,-6.246)**	**-1.271(-1.894,-0.648)**	**-0.761(-1.381,-0.141)**	**-0.912(-1.511,-0.313)**	
3-5 days/week	**-7.794(-8.533,-7.055)**	**-1.265(-1.933,-0.596)**	**-0.824(-1.490,-0.159)**	**-1.171(-1.814,-0.527)**	
6-7 days/week	**-5.529(-6.111,-4.946)**	**-0.601(-1.129,-0.074)**	**-0.572(-1.101,-0.043)**	**-1.064(-1.576,-0.552)**	
Males^a^					
No	Ref	Ref	Ref	Ref	0.153
<1 day/week	**-2.414(-3.563,-1.265)**	-0.348(-1.428,0.733)	-0.191(-1.263,0.881)	-0.269(-1.306,0.768)	
1-2 days/week	**-4.681(-5.662,-3.700)**	**-0.965(-1.899,-0.031)**	-0.625(-1.556,0.306)	**-0.931(-1.832,-0.030)**	
3-5 days/week	**-5.234(-6.264,-4.203)**	-0.714(-1.702,0.274)	-0.371(-1.360,0.618)	-0.847(-1.805,0.110)	
6-7 days/week	**-3.997(-4.812,-3.182)**	-0.301(-1.083,0.480)	-0.349(-1.142,0.443)	-0.676(-1.444,0.092)	
Females^a^					
No	Ref	Ref	Ref	Ref	**<0.001**
<1 day/week	**-4.865(-5.953,-3.777)**	-0.451(-1.377,0.476)	-0.146(-1.066,0.773)	-0.319(-1.213,0.574)	
1-2 days/week	**-8.551(-9.503,-7.599)**	**-1.190(-2.013,-0.367)**	-0.635(-1.455,0.184)	-0.681(-1.478,0.116)	
3-5 days/week	**-10.011(-11.041,-8.981)**	**-1.474(-2.367,-0.581)**	**-1.044(-1.933,-0.156)**	**-1.251(-2.115,-0.388)**	
6-7 days/week	**-7.087(-7.898,-6.276)**	**-0.732(-1.435,-0.029)**	-0.681(-1.382,0.020)	**-1.215(-1.897,-0.534)**	
Strength of spicy food consumption					
Total					
No	Ref	Ref	Ref	Ref	**<0.001**
Weak	**-4.360(-4.819,-3.901)**	**-0.640(-1.054,-0.227)**	**-0.500(-0.912,-0.088)**	**-0.788(-1.186,-0.389)**	
Moderate	**-5.117(-5.711,-4.524)**	**-0.496(-1.034,0.042)**	**-0.678(-1.219,-0.137)**	**-1.225(-1.748,-0.701)**	
Strong	**-1.794(-3.109,-0.478)**	**-0.305(-1.468,0.859)**	-0.847(-2.004,0.311)	**-1.444(-2.563,-0.325)**	
Males^a^					
No	Ref	Ref	Ref	Ref	0.113
Weak	**-3.283(-3.937,-2.629)**	-0.507(-1.132,0.118)	-0.395(-1.023,0.232)	**-0.639(-1.247,-0.032)**	
Moderate	**-3.584(-4.373,-2.795)**	0.169(-0.588,0.925)	-0.045(-0.816,0.726)	-0.579(-1.326,0.168)	
Strong	-1.476(-3.166,0.214)	-0.202(-1.785,1.380)	-0.600(-2.185,0.985)	-0.980(-2.514,0.554)	
Females^a^					
No	Ref	Ref	Ref	Ref	**<0.001**
Weak	**-5.239(-5.868,-4.611)**	**-0.646(-1.188,-0.104)**	-0.522(-1.061,0.018)	**-0.788(-1.313,-0.263)**	
Moderate	**-8.099(-8.976,-7.222)**	**-1.284(-2.042,-0.526)**	**-1.389(-2.145,-0.633)**	**-1.807(-2.542,-1.072)**	
Strong	**-2.991(-4.980,-1.003)**	0.087(-1.597,1.771)	-0.544(-2.219,1.131)	-1.310(-2.939,0.319)	
Years of eating spicy food-to-age ratio					
Total	**5.929(4.773,7.086)**	**-4.333(-5.373,-3.293)**	**-4.383(-5.412,-3.355)**	**-4.538(-5.530,-3.546)**	**<0.001**
Males^a^	**4.340(2.748,5.932)**	**-3.344(-4.866,-1.821)**	**-3.313(-4.824,-1.801)**	**-2.952(-4.413,-1.492)**	**<0.001**
Females^a^	**7.611(5.986,9.236)**	**-5.195(-6.594,-3.795)**	**-5.419(-6.802,-4.036)**	**-5.853(-7.195,-4.512)**	**<0.001**

Model 1: original model without any adjustments

Model 2: adjusted for age, gender (male or female), household income (< 12 000 yuan, 12 000–19 999 yuan, 20 000–59 999 yuan,60 000–99 999 yuan, ≥ 100 000 yuan), family history of hypertension (yes or no)

Model 3: adjusted for model 2 plus smoking status (never, former, and current), alcohol drinking (continuous), physical activity (continuous), total energy intake per day (continuous), DASH score (continuous), snoring (no, occasionally, habitual)

Model 4: adjusted for model 3 plus BMI (continuous), waist circumference (continuous), dyslipidemia (yes or no), diabetes (yes or no)

^a^without adjustment for gender

### Association between spicy food consumption and DBP

A positive association was observed between the years of eating spicy food-to-age ratio and DBP in males, with a corresponding β coefficient (95% CI) of 1.300 (0.338, 2.263), whereas neither the frequency nor the strength of spicy food consumption was found to be significant (Table 4). In females, there was almost no association between consuming spicy food and DBP (all *P* trends > 0.05), except for a decrease of 0.591 mmHg (95%CI: -1.078, -0.105) in DBP among participants who consumed spicy food 1–2 days/week, compared to those who did not consume spicy food.

**Table 4:** Association between spicy food consumption and DBP

	*β* coefficients (95%*CI*)				*P* trend for model 4
	Model 1	Model 2	Model 3	Model 4	
Frequency of spicy food consumption					
Total					
No	Ref	Ref	Ref	Ref	0.841
<1 day/week	**-0.515(-0.978,-0.051)**	0.111(-0.332,0.555)	0.474(-0.224,1.172)	0.057(-0.369,0.483)	
1-2 days/week	**-1.149(-1.550,-0.747)**	-0.278(-0.668,0.111)	0.387(-0.220,0.994)	-0.277(-0.652,0.099)	
3-5 days/week	**-0.705(-1.134,-0.277)**	0.124(-0.294,0.541)	0.599(-0.046,1.243)	-0.094(-0.498,0.309)	
6-7 days/week	0.093(-0.246,0.431)	0.602(0.272,0.931)	0.638(0.121,1.154)	0.053(-0.267,0.374)	
Males^a^					
No	Ref	Ref	Ref	Ref	0.203
<1 day/week	0.560(-0.149,1.269)	0.577(-0.130,1.283)	-0.189(-0.734,0.356)	0.409(-0.266,1.084)	
1-2 days/week	0.449(-0.156,1.054)	0.483(-0.128,1.093)	**-0.591(-1.078,-0.105)**	0.161(-0.426,0.748)	
3-5 days/week	**0.776(0.140,1.412)**	**0.799(0.153,1.445)**	-0.334(-0.861,0.193)	0.232(-0.391,0.856)	
6-7 days/week	**1.062(0.560,1.565)**	**1.135(0.624,1.646)**	-0.159(-0.575,0.258)	0.371(-0.129,0.871)	
Females^a^					
No	Ref	Ref	Ref	Ref	0.915
<1 day/week	**-1.061(-1.637,-0.486)**	-0.186(-0.747,0.375)	-0.127(-0.685,0.431)	-0.189(-0.734,0.356)	
1-2 days/week	**-2.241(-2.744,-1.737)**	**-0.727(-1.226,-0.229)**	**-0.571(-1.068,-0.073)**	-**0.591(-1.078,-0.105)**	
3-5 days/week	**-2.068(-2.613,-1.524)**	-0.325(-0.866,0.216)	-0.243(-0.782,0.296)	-0.334(-0.861,0.193)	
6-7 days/week	**-1.067(-1.496,-0.638)**	0.221(-0.205,0.646)	0.122(-0.303,0.548)	-0.159(-0.575,0.258)	
Strength of spicy food consumption					
Total					
No	Ref	Ref	Ref	Ref	0.231
Weak	**-0.373(-0.639,-0.107)**	0.125(-0.134,0.383)	0.042(-0.215,0.300)	-0.130(-0.380,0.120)	
Moderate	**0.897(0.553,1.241)**	**0.909(0.573,1.245)**	**0.530(0.192,0.868)**	0.190(-0.139,0.518)	
Strong	**1.573(0.811,2.335)**	**1.178(0.451,1.904)**	0.578(-0.146,1.301)	0.210(-0.492,0.912)	
Males^a^					
No	Ref	Ref	Ref	Ref	0.094
Weak	0.393(-0.01,0.797)	**0.447(0.038,0.855)**	0.227(-0.182,0.636)	0.037(-0.358,0.433)	
Moderate	**1.407(0.921,1.894)**	**1.489(0.994,1.983)**	**0.890(0.387,1.392)**	0.469(-0.018,0.955)	
Strong	**1.140(0.099,2.182)**	**1.139 (0.105,2.173)**	0.428(-0.605,1.460)	0.065(-0.934,1.064)	
Females^a^					
No	Ref	Ref	Ref	Ref	0.889
Weak	**-1.032(-1.364,-0.700)**	-0.074(-0.403,0.254)	-0.082(-0.409,0.246)	-0.234(-0.554,0.086)	
Moderate	**-1.113(-1.576,-0.649)**	0.307(-0.152,0.766)	0.143(-0.317,0.602)	-0.080(-0.529,0.369)	
Strong	0.987(-0.064,2.038)	**1.533(0.513,2.553)**	**1.058(0.041,2.075)**	0.627(-0.367,1.621)	
Years of eating spicy food-to-age ratio					
Total	**2.664(1.984,3.344)**	**1.158(0.499,1.817)**	**0.953(0.301,1.605)**	**0.838(0.207,1.469)**	**0.009**
Males^a^	**1.844(0.850,2.838)**	**1.410(0.400,2.419)**	**1.129(0.131,2.128)**	**1.300(0.338,2.263)**	**0.008**
Females^a^	**3.723(2.856,4.589)**	**0.926(0.068,1.785)**	0.749(-0.103,1.601)	0.493(-0.338,1.325)	0.245

Model 1: original model without any adjustments

Model 2: adjusted for age, gender (male or female), household income (< 12 000 yuan, 12 000–19 999 yuan, 20 000–59 999 yuan,60 000–99 999 yuan, ≥ 100 000 yuan), family history of hypertension (yes or no)

Model 3: adjusted for model 2 plus smoking status (never, former, and current), alcohol drinking (continuous), physical activity (continuous), total energy intake per day (continuous), DASH score (continuous), snoring (no, occasionally, habitual)

Model 4: adjusted for model 3 plus BMI (continuous), waist circumference (continuous), dyslipidemia (yes or no), diabetes (yes or no)

^a^without adjustment for gender

### Subgroup analyses

**Fig. 1:**
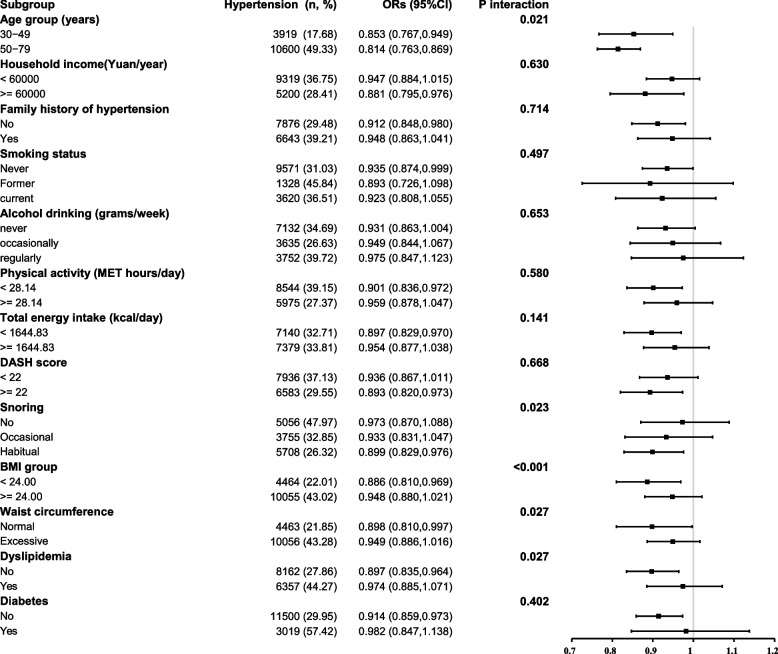
Subgroup analyses of the association between eating spicy food and hypertension

Figure 1 shows the subgroup analyses. The association between spicy food consumption ( no VS yes) and hypertension was largely consistent across subgroups defined by household income, family history of hypertension, smoking status, alcohol drinking, physical activity, total energy intake, DASH score, and diabetes (*P* interaction > 0.05). However, a significantly stronger association in subgroup participants aged 50–79 years, who had habitual snoring, a BMI < 24.00 kg/m^2^, a normal waist circumference, and no dyslipidemia. The corresponding adjusted ORs (95%CIs) were 0.814 (0.763, 0.869), 0.899 (0.829, 0.976), 0.886 (0.810, 0.969), 0.898 (0.810, 0.997), and 0.897 (0.835, 0.964), respectively.

### Sensitivity analyses

Sensitivity analyses showed that associations of spicy food intake with hypertension or blood pressure did not change markedly after excluding 1,551 participants with self-reported peptic ulcer disease diagnosed by a physician (Supplementary Table 1). For hypertension, compared with participants who do not eat spicy food, ORs (95% CIs) of those who consume spicy food 6–7 days/week, who consume spicy food with strong strength, and years of eating spicy food-to-age ratio among females were 0.902 (0.812, 1.002), 0.765 (0.592, 0.989), and 0.637 (0.507, 0.802), respectively. For SBP, compared with the reference group, β coefficients (95% CIs) of 1–2 days/week, 3–5 days/week, 6–7 days/week, weak strength, and years of eating spicy food-to-age ratio among males were − 1.067 (-1.992, -0.142), -1.032 (-2.013, -0.051), -0.800 (-1.588, -0.012), -0.674 (-1.297, -0.052), and − 2.986 (-4.481, -1.492), respectively; and β coefficients (95% CIs) of 3–5 days/week, 6–7 days/week, weak strength, moderate strength and years of eating spicy food-to-age ratio among females were − 1.196 (-2.076, -0.315), -1.171 (-1.868, -0.474), -0.740 (-1.275, -0.206), -1.764 (-2.511, -1.018), and − 5.852 (-7.213,-4.491), respectively. For DBP, β coefficients (95% CIs) of years of eating spicy food-to-age ratio among males was 1.362 (0.377, 2.347), and β coefficients (95% CIs) of consuming spicy food 1–2 days/week among females was − 0.528 (-1.024,-0.032), respectively.

## Discussion

In line with previous studies [17, 28], participants who ate spicy food more frequently ate spicier, started to eat earlier, and had a greater years of eating spicy food to age ratio. We also found that participants who consumed spicy food more frequently were more likely to be younger, male, current smokers, and to have a family history of hypertension; to drink more alcohol; to have higher household income, higher total energy intake, higher DASH scores, and higher levels of physical activity; but were less likely to be snorers. Compared to other studies, the links between age, gender, smoking, drinking, and energy intake and spicy food consumption are almost consistent with this study [14, 24, 25]. However, household income [24, 28], snoring [24], physical activity [24], and BMI [14, 21, 24, 25] were found to have no significant difference or even an opposite association with spicy food consumption in other studies. These differences may be related to variations in spicy food consumption habits across different regions. Wang et al. [40] and Zhao et al. [16] both found that spicy food preference was concentrated in Sichuan and Chongqing areas. In the present study, 79.3% of residents living in the Sichuan Basin ate spicy food weekly with 2.06% preferring strong pungency, and more than half of them (51.3%) consumed chili almost every day, whereas only 42.5% consumed spicy food weekly for the CKB population, 12.3% of Zhejiang residents, and 8.8% of Haikou people [8, 24]. These findings indicate that spicy eating habits vary widely in different regions. Further research is needed to explore the relationships between spicy food consumption and demographic characteristics such as household income, lifestyle factors such as physical activity and snoring, as well as body mass index.

To our knowledge, this is the first study to combine the intake frequency and strength of spicy food and the years of eating spicy food to age ratio in exploring the association between spicy food consumption and hypertension in the Sichuan Basin. In addition, we specifically investigated the effects of spicy food consumption on both systolic and diastolic blood pressure separately. For hypertension prevalence, the frequency and strength of spicy food intake as well as the years of eating spicy food-to-age ratio were all inversely correlated with hypertension among females but not among males. Compared to non-consumers, females who consumed spicy food 6–7 days/week had an 11.4% lower risk of developing hypertension, while those who consumed spicy food with strong strength had a 24.3% lower risk. As the years of eating spicy food-to-age ratio increased by one unit, female participants experienced a 36.8% decrease in the risk of developing hypertension. These findings were almost consistent with the other two cross-sectional studies [24, 25]. Wang et al. [24] found that females who consumed spicy food ≥ 3 times weekly had a 22% lower risk of hypertension, but no significant association was found in males. He et al. [25] also found that there was a 36% decrease in females who consumed spicy food usually and a 34% decrease in females who ate spicy food with moderate pungency. However, unlike the previous two studies, our study also found an inverse correlation between spicy food consumption and SBP in men, which may be due to three reasons: firstly, the effect of spicy food on hypertension may indeed be smaller in men compared to women. In the current study, for every unit increased in the years of eating spicy food to age ratio, SBP decreased by 2.952 mmHg in men, while it decreased by 5.853 mmHg in women. Secondly, the previous studies included participants from different regions in China, with varying proportions of spicy food consumption, and the overall proportion of consuming spicy food was much lower than that in the Sichuan Basin. Lastly, in the previous two studies, the sample size of men may not be large enough to find a difference. A prospective cohort study [26] showed that people with increasing cumulative average chili intake were less likely to develop hypertension. Several animal experiments also demonstrated that capsaicin (the major pungent ingredient in spicy food) feeding had an antihypertensive effect in rats genetically predisposed to hypertension and mice induced by a high-salt diet [41–43]. The above evidence suggests that spicy food can reduce SBP and has an antihypertensive effect, especially in women. It’s biologically plausible: capsaicin, as the major pungent element in chili, is a neurotoxic agent and could activate transient receptor potential vanilloid type-1 (TRPV1), in turn increasing the expression phosphorylation of protein kinase A (PKA) and endothelial nitric oxide synthase (eNOS), thus, lowering blood pressure [43–46].

It was also the first study to explore the association between the years of eating spicy food-to-age ratio and DBP. In contrast to SBP levels, the years of eating spicy food-to-age ratio appeared to be positively correlated with DBP in men, whereas neither the frequency nor the strength of spicy food consumption was found to be significant. In females, there was almost no association between consuming spicy food and DBP, except for a decrease of 0.591 mmHg (95%CI: -1.078, -0.105) in DBP among participants who consumed spicy food 1–2 days/week when compared to non-consumers. However, the frequency of spicy food consumption was found to be negatively related to DBP (β, 95%CI: -0.57, -1.08, -0.05) among females living in Zhejiang [24]. In the China Health and Nutrition Survey conducted in 2009, higher frequency (≥ 5 times/week VS 0 time/week, β, 95%CI: -1.863, -2.870, -0.856) and strength (moderate pungency VS non-eating, β, 95%CI: -1.349, -2.114, -0.584) of spicy food consumption were both significantly associated with lower DBP in women [25]. According to the findings above, both the frequency and strength of consuming spicy food have a similar effect on DBP demonstrating a protective effect on DBP among females but not among males. However, this study also investigated the years of eating spicy food-to-age ratio and found a positive association with DBP only in males but not in females. These findings suggest a gender difference in the effect of spicy food on DBP, with a possible decrease in DBP among females who consume spicy food 1–2 days/week, and a potential increase in DBP among males who consume spicy food.

In the stratified analyses, participants in the subgroup who were 50 to 79 years old (OR, 95%CI: 0.814, 0.763, 0.869), habitually snored (OR, 95%CI: 0.899, 0.829, 0.976), had a BMI < 24 kg/m^2^ (OR, 95%CI: 0.886, 0.810, 0.969), had a normal waist circumference (OR, 95%CI: 0.898, 0.810, 0.997), and had no dyslipidemia (OR, 95%CI: 0.897, 0.835, 0.964) showed a significantly stronger association. However, in the subgroup analyses conducted by Wang et al. [24], they only found significant differences between non-current drinkers (OR, 95%CI: 0.72, 0.62, 0.84) and current drinkers (OR, 95%CI: 0.98, 0.80, 1.20). They did not conduct stratified analyses based on snoring habits or the presence of dyslipidemia and the included covariates in their analysis differed from the current study: they did not consider the influence of dyslipidemia and family history of hypertension; dietary factors were only considered whether meat and fruits were consumed daily or not; additionally, the participants were from different regions with different lifestyles and risk factors, which may have contributed to the differences. Overall, the protective effect of spicy food consumption on hypertension appears to be greater in individuals with fewer risk factors.

Some limitations should be noted in this study. Firstly, cross-sectional studies cannot establish a causal relationship between consuming spicy food and hypertension. Further experimental or prospective research is needed to confirm this. Secondly, spicy food consumption is self-reported based on past experiences, which introduces recall bias, and the assessment of the strength of spiciness is subject, leading to measurement errors. Therefore, it is necessary to develop tools or methods that can objectively judge spicy food consumption in the future. Thirdly, although we adjusted for major socioeconomic and lifestyle factors and clinical variables in the multivariable analyses, there may still be residual confounding factors that cannot be measured or are unknown. The E-values for the association between years of eating spicy food-to-age ratio and hypertension in females, the association between years of eating spicy food-to-age ratio and SBP in females, and the association between years of eating spicy food-to-age ratio and DBP in males were 1.83, 4.82, and 21.74, respectively. Lastly, these results were obtained from Han Chinese residents aged 30–79 years, from regions eating spicy foods relatively often, so extrapolating the findings to other areas or populations should be noted.

## Conclusions

In conclusion, spicy food consumption may be negatively correlated with the prevalence of hypertension in women but not in men. The protective effect of spicy food on hypertension seems to be stronger in individuals who are 50 to 79 years old, habitually snored, have a BMI < 24 kg/m^2^, have a normal waist circumference, and have no dyslipidemia. For SBP, spicy food consumption also shows a protective effect on SBP in both genders, although the effect seems to be smaller in males compared to females. In terms of DBP, gender differences still exist, as spicy food consumption may decrease it in women but increase it in men. Overall, our findings provide evidence that spicy food consumption might be a choice to reduce hypertension risk by decreasing SBP, especially for females and individuals with fewer risk factors. Further multicenter prospective cohort studies are needed to further confirm the potential role of spicy food in the regulation of blood pressure.

### Supplementary Information

**Additional file 1: Supplementary Table 1.** Sensitivity analyses on the association between spicy food consumption and hypertension, SBP, and DBP.

## Data Availability

Our study relied on data from the China Multi-Ethnic Cohort study. The summary dataset used and analyzed during the current study is available from the corresponding author upon reasonable request.

## Abbreviations

CMEC: China Multi-Ethnic Cohort
DASH score: Dietary Approaches to Stop Hypertension (DASH) diet score
MET: Metabolic equivalent of task
BMI: Body mass index
SBP: Systolic blood pressure
DBP: Diastolic blood pressure
FFQ: Food frequency questionnaire
CHNS: China Health and Nutrition Survey
OR: Odds ratio
CI: Confidence interval
TRPV1: Transient receptor potential vanilloid type-1
PKA: Phosphorylation of protein kinase A
eNOS: Endothelial nitric oxide synthase

## References

[1] Lacey B, Lewington S, Clarke R, Kong XL, Chen Y, Guo Y, Yang L, Bennett D, Bragg F, Bian Z (2018). Age-specific association between blood pressure and vascular and non-vascular chronic diseases in 0.5 million adults in China: a prospective cohort study. Lancet Global Health.

[2] Ettehad D, Emdin CA, Kiran A, Anderson SG, Callender T, Emberson J, Chalmers J, Rodgers A, Rahimi K (2016). Blood pressure lowering for prevention of cardiovascular disease and death: a systematic review and meta-analysis. Lancet.

[3] Murray CJL, Aravkin AY, Zheng P, Abbafati C, Abbas KM, Abbasi-Kangevari M, Abd-Allah F, Abdelalim A, Abdollahi M, Abdollahpour I (2020). Global burden of 87 risk factors in 204 countries and territories, 1990–2019: a systematic analysis for the global burden of Disease Study 2019. Lancet.

[4] Wang Z, Chen Z, Zhang L, Wang X, Hao G, Zhang Z, Shao L, Tian Y, Dong Y, Zheng C (2018). Status of hypertension in China results from the China Hypertension Survey, 2012–2015. Circulation.

[5] Nguyen H, Odelola OA, Rangaswami J, Amanullah A (2013). A review of nutritional factors in hypertension management. Int J Hypertens.

[6] Ao Z, Huang Z, Liu H. Spicy food and chili peppers and multiple health outcomes: umbrella review. Mol Nutr Food Res. 2022;66(23):e2200167. 10.1002/mnfr.202200167 PMC10078540 36111960

[7] Bonaccio M, Di Castelnuovo A, Costanzo S, Ruggiero E, De Curtis A, Persichillo M, Tabolacci C, Facchiano F, Cerletti C, Donati MB (2019). Chili Pepper Consumption and Mortality in italian adults. J Am Coll Cardiol.

[8] Lv J, Qi L, Yu C, Yang L, Guo Y, Chen Y, Bian Z, Sun D, Du J, Ge P (2015). Consumption of spicy foods and total and cause specific mortality: population based cohort study. BMJ.

[9] Dong X, Li Y, Yang K, Zhang L, Xue Y, Yu S, Liu X, Tu R, Qiao D, Luo Z (2021). Associations of spicy food flavour and intake frequency with blood lipid levels and risk of abnormal serum lipid levels in chinese rural population: a cross-sectional study. Public Health Nutr.

[10] Kelava L, Nemeth D, Hegyi P, Keringer P, Kovacs DK, Balasko M, Solymar M, Pakai E, Rumbus Z, Garami A. Dietary supplementation of transient receptor potential vanilloid-1 channel agonists reduces serum total cholesterol level: a meta-analysis of controlled human trials. Crit Rev Food Sci Nutr. 2022;62(25):7025–35. 10.1080/10408398.2021.1910138 33840333

[11] Wen QR, Liu Q, Lyu J, Guo Y, Pei P, Yang L, Du HD, Chen YP, Chen JS, Yu CQ (2022). Spicy food consumption and risk of lip, oral cavity and pharynx cancers: a prospective cohort study of chinese adults. Zhonghua Liuxingbingxue Zazhi.

[12] Chan WC, Millwood IY, Kartsonaki C, Du H, Guo Y, Chen Y, Bian Z, Walters RG, Lv J, He P (2021). Spicy food consumption and risk of gastrointestinal-tract cancers: findings from the China Kadoorie Biobank. Int J Epidemiol.

[13] Mosqueda-Solis A, Lafuente-Ibanez de Mendoza I, Manuel Aguirre-Urizar J, Mosqueda-Taylor A (2021). Capsaicin intake and oral carcinogenesis: a systematic review. Med Oral Patologia Oral Y Cir Bucal.

[14] Xue Y, He T, Yu K, Zhao A, Zheng W, Zhang Y, Zhu B (2017). Association between spicy food consumption and lipid profiles in adults: a nationwide population-based study. Br J Nutr.

[15] Yu K, Xue Y, He T, Guan L, Zhao A, Zhang Y (2018). Association of Spicy Food Consumption frequency with serum lipid profiles in older people in China. J Nutr Health Aging.

[16] Zhao Z, Li M, Li C, Wang T, Xu Y, Zhan Z, Dong W, Shen Z, Xu M, Lu J (2020). Dietary preferences and diabetic risk in China: a large-scale nationwide internet data-based study. J Diabetes.

[17] Sun D, Lv J, Chen W, Li S, Guo Y, Bian Z, Yu C, Zhou H, Tan Y, Chen J (2014). Spicy food consumption is associated with adiposity measures among half a million chinese people: the China Kadoorie Biobank study. BMC Public Health.

[18] Yang X, Tang W, Mao D, Liu X, Qian W, Dai Y, Chen L, Ding X (2022). Spicy food consumption is associated with abdominal obesity among chinese Han population aged 30–79 years in the Sichuan Basin: a population-based cross-sectional study. BMC Public Health.

[19] Yang K, Li Y, Mao Z, Liu X, H Zhang, Liu R, Xue Y, Tu R, Liu X, Zhang X (2018). Relationship between spicy flavor, spicy food intake frequency, and general obesity in a rural adult chinese population: the RuralDiab study. Nutr, Metabol, Cardiovasc Dis: NMCD..

[20] Luo Q, Ding R, Chen L, Bu X, Xiao M, Liu X, Wu Y, Xu J, Tang W, Qiu J (2022). The Association between Spicy Food Intake and Risk of Hyperuricemia among chinese adults. Front Public Health.

[21] Dong X, Li Y, Yang K, Zhang L, Xue Y, Yu S, Liu X, Tu R, Qiao D, Luo Z (2020). Mediation effect of body mass index on the association between spicy food intake and hyperuricemia in rural chinese adults: the Henan rural cohort study. BMC Public Health.

[22] Xie P, Xia W, Lowe S, Zhou Z, Ding P, Cheng C, Bentley R, Li Y, Wang Y, Zhou Q (2022). High spicy food intake may increase the risk of esophageal cancer: a meta-analysis and systematic review. Nutr Res (New York NY).

[23] Shirani F, Foshati S, Tavassoly M, Clark CCT, Rouhani MH (2021). The effect of red pepper/capsaicin on blood pressure and heart rate: a systematic review and meta-analysis of clinical trials. Phytother Res.

[24] Wang H, Chen L, Shen D, Cao Y, Zhang X, Xie K, Wang C, Zhu S, Pei P, Guo Y (2021). Association between frequency of spicy food consumption and hypertension: a cross-sectional study in Zhejiang Province, China. Nutr Metab (Lond).

[25] He T, Wang M, Tian Z, Zhang J, Liu Y, Zhang Y, Wang P, Xue Y (2019). Sex-dependent difference in the association between frequency of spicy food consumption and risk of hypertension in chinese adults. Eur J Nutr.

[26] Shi Z, Riley M, Brown A, Page A (2018). Chilli intake is inversely associated with hypertension among adults. Clin Nutr ESPEN.

[27] Harada N, Okajima K (2009). Effects of Capsaicin and Isoflavone on blood pressure and serum levels of insulin-like Growth Factor-I in Normotensive and Hypertensive volunteers with Alopecia. Bioscience Biotechnol Biochem.

[28] Wen Q, Wei Y, Du H, Lv J, Guo Y, Bian Z, Yang L, Chen Y, Chen Y, Shi L (2021). Characteristics of spicy food consumption and its relation to lifestyle behaviours: results from 0.5 million adults. Int J Food Sci Nutr.

[29] Astrup A, Kristensen M, Gregersen NT, Belza A, Lorenzen JK, Due A, Larsen TM (2010). Can bioactive foods affect obesity?. Ann N Y Acad Sci.

[30] Zhao X, Hong F, Yin J, Tang W, Zhang G, Liang X, Li J, Cui C, Li X (2021). China multi-ethnic cohort collaborative g: Cohort Profile: the China multi-ethnic cohort (CMEC) study. Int J Epidemiol.

[31] China Hypertension Prevention Guideline Revision Committee (2019). Chinese guidelines for the Prevention and Treatment of Hypertension (2018 revision). Chin J Cardiol.

[32] Chobanian AV, Bakris GL, Black HR, Cushman WC, Green LA, Izzo JL, Jones DW, Materson BJ, Oparil S, Wright JT (2003). The Seventh Report of the Joint National Committee on Prevention, detection, evaluation, and treatment of high blood pressure: the JNC 7 report. JAMA.

[33] Ainsworth BE, Haskell WL, Whitt MC, Irwin ML, Swartz AM, Strath SJ, O’Brien WL, Bassett DR, Schmitz KH, Emplaincourt PO (2000). Compendium of physical activities: an update of activity codes and MET intensities. Med Sci Sports Exerc.

[34] Xiao X, Qin Z, Lv X, Dai Y, Ciren Z, Yangla Y, Zeng P, Ma Y, Li X, Wang L (2021). Dietary patterns and cardiometabolic risks in diverse less-developed ethnic minority regions: results from the China multi-ethnic cohort (CMEC) study. Lancet Reg Health Western Pac.

[35] Millwood IY, Walters RG, Mei XW, Guo Y, Yang L, Bian Z, Bennett DA, Chen Y, Dong C, Hu R (2019). Conventional and genetic evidence on alcohol and vascular disease aetiology: a prospective study of 500 000 men and women in China. Lancet.

[36] National Health Commission of the People’s Republic of China. Dietary guide for adult diabetes patients (WS/T 429–2013). 2013. https://down.foodmate.net/standard/sort/16/68048.html.

[37] National Institute for Nutrition and Health. China food composition tables. 6th ed. Beijing: Peking university medical press; 2018.

[38] Chinese Joint Committee on the Revision of Guidelines for Prevention and Treatment of Adult Dyslipidemia (2016). Guidelines for prevention and treatment of dyslipidemia in chinese adults (2016 version). Chin Circ J.

[39] Association AD (2016). 2. Classification and diagnosis of diabetes. Diabetes Care.

[40] Wang S, Cheng L, He S, Xie D (2019). Regional Pungency Degree in China and its correlation with typical climate factors. J Food Sci.

[41] Li L, Wang F, Wei X, Liang Y, Cui Y, Gao F, Zhong J, Pu Y, Zhao Y, Yan Z (2014). Transient receptor potential vanilloid 1 activation by dietary capsaicin promotes urinary sodium excretion by inhibiting epithelial sodium channel α subunit-mediated sodium reabsorption. Hypertension.

[42] Hao X, Chen J, Luo Z, He H, Yu H, Ma L, Ma S, Zhu T, Liu D, Zhu Z (2011). TRPV1 activation prevents high-salt diet-induced nocturnal hypertension in mice. Pflug Arch: Eur J Physiol.

[43] Yang D, Luo Z, Ma S, Wong WT, Ma L, Zhong J, He H, Zhao Z, Cao T, Yan Z (2010). Activation of TRPV1 by Dietary Capsaicin improves endothelium-dependent vasorelaxation and prevents hypertension. Cell Metabol.

[44] Xu X, Wang P, Zhao Z, Cao T, He H, Luo Z, Zhong J, Gao F, Zhu Z, Li L (2011). Activation of transient receptor potential vanilloid 1 by dietary capsaicin delays the onset of stroke in stroke-prone spontaneously hypertensive rats. Stroke.

[45] Hollis M, Wang DH (2013). Transient receptor potential vanilloid in blood pressure regulation. Curr Opin Nephrol Hypertens.

[46] Zhang M-J, Yin Y-W, Li B-H, Liu Y, Liao S-Q, Gao C-Y, Li J-C, Zhang L-L (2015). The role of TRPV1 in improving VSMC function and attenuating hypertension. Prog Biophys Mol Biol.

