# Supplementary material for: Association between spicy food and hypertension among Han Chinese aged 30–79 years in Sichuan Basin: a population-based cross-sectional study
Source: BMC Public Health. 2023 Aug 30;23:1663. doi: 10.1186/s12889-023-16588-6 (PMC10466726; doi:10.1186/s12889-023-16588-6)
Supplement: Supplementary file 1 — Additional file 1: Supplementary Table 1. Sensitivity analyses on the association between spicy food consumption and hypertension, SBP, and DBP. [file 12889_2023_16588_MOESM1_ESM.docx]

**Supplementary Table 1 Sensitivity analyses on the association between spicy food consumption and hypertension, SBP, and DBP**

|  |  | Hypertension |  |  | SBP | |  | DBP | |
| --- | --- | --- | --- | --- | --- | --- | --- | --- | --- |
|  |  | ORs (95%CIs) | *P* trend |  | β coefficients (95%CIs) | *P* trend |  | β coefficients (95%CIs) | *P* trend |
| Frequency of spicy food consumption | |  |  |  |  |  |  |  |  |
| Total |  |  | **0.807** |  |  | **<0.001** |  |  | 0.354 |
|  | No | Ref |  |  | Ref |  |  | Ref |  |
|  | <1 day/week | 0.964(0.871,1.067) |  |  | -0.445(-1.139,0.249) | |  | 0.019(-0.416,0.454) |  |
|  | 1-2 days/week | **0.901(0.823,0.987)** |  |  | **-0.920(-1.533,-0.307)** | |  | -0.280(-0.665,0.104) |  |
|  | 3-5 days/week | 0.923(0.836,1.018) |  |  | **-1.204(-1.862,-0.546)** | |  | -0.116(-0.528,0.296) |  |
|  | 6-7 days/week | **0.925(0.857,0.997)** |  |  | **-1.085(-1.610,-0.561)** | |  | 0.054(-0.275,0.382) |  |
| Males* |  |  | 0.753 |  |  | 0.125 |  |  | 0.273 |
|  | No | Ref |  |  | Ref |  |  | Ref |  |
|  | <1 day/week | 1.021(0.880,1.185) |  |  | -0.475(-1.536,0.586) |  |  | 0.265(-0.426,0.956) |  |
|  | 1-2 days/week | 0.923(0.810,1.051) |  |  | **-1.067(-1.992,-0.142)** | |  | 0.063(-0.539,0.666) |  |
|  | 3-5 days/week | 0.954(0.831,1.096) |  |  | **-1.032(-2.013,-0.051)** | |  | 0.112(-0.527,0.751) |  |
|  | 6-7 days/week | 0.976(0.874,1.089) |  |  | **-0.800(-1.588,-0.012)** | |  | 0.285(-0.228,0.799) |  |
| Females* |  |  |  |  |  | **<0.001** |  |  | 0.717 |
|  | No | Ref | 0.093 |  | Ref |  |  | Ref |  |
|  | <1 day/week | 0.934(0.812,1.075) |  |  | -0.315(-1.226,0.596) |  |  | -0.148(-0.704,0.409) |  |
|  | 1-2 days/week | 0.905(0.796,1.028) |  |  | -0.625(-1.437,0.188) | |  | **-0.528(-1.024,-0.032)** |  |
|  | 3-5 days/week | 0.917(0.796,1.057) |  |  | **-1.196(-2.076,-0.315)** | |  | -0.286(-0.823,0.252) |  |
|  | 6-7 days/week | **0.902(0.812,1.002)** |  |  | **-1.171(-1.868,-0.474)** | |  | -0.097(-0.522,0.329) |  |
| Strength of spicy food consumption | |  |  |  |  |  |  |  |  |
| Total |  |  |  |  |  | **<0.001** |  |  | 0.249 |
|  | No | Ref | **0.045** |  | Ref |  |  | Ref |  |
|  | Weak | **0.934(0.879,0.991)** |  |  | **-0.763(-1.170,-0.356)** | |  | -0.111(-0.366,0.144) |  |
|  | Moderate | 0.958(0.885,1.038) |  |  | **-1.231(-1.765,-0.698)** | |  | 0.174(-0.161,0.508) |  |
|  | Strong | **0.809(0.685,0.956)** |  |  | **-1.466(-2.602,-0.330)** | |  | 0.247(-0.465,0.959) |  |
| Males* |  |  |  |  |  | 0.079 |  |  | 0.144 |
|  | No | Ref | 0.719 |  |  |  |  | Ref |  |
|  | Weak | 0.943(0.864,1.030) |  |  | **-0.674(-1.297,-0.052)** | |  | 0.012(-0.394,0.417) |  |
|  | Moderate | 0.997(0.895,1.111) |  |  | -0.636(-1.401,0.128) | |  | 0.414(-0.084,0.912) |  |
|  | Strong | 0.888(0.713,1.106) |  |  | -1.155(-2.725,0.414) |  |  | 0.049(-0.973,1.070) |  |
| Females* |  |  |  |  |  | **<0.001** |  |  | 0.768 |
|  | No | Ref | **0.030** |  | Ref |  |  | Ref |  |
|  | Weak | 0.940(0.865,1.022) |  |  | **-0.740(-1.275,-0.206)** | |  | -0.189(-0.515,0.138) |  |
|  | Moderate | 0.916(0.811,1.035) |  |  | **-1.764(-2.511,-1.018)** | |  | -0.062(-0.518,0.393) |  |
|  | Strong | **0.765(0.592,0.989)** |  |  | -1.193(-2.833,0.446) |  |  | 0.691(-0.311,1.692) |  |
| Years of eating spicy food-to-age ratio | |  |  |  |  |  |  |  |  |
|  | Total | **0.720(0.614,0.844)** | **<0.001** |  | **-4.552(-5.562,-3.541)** | **<0.001** |  | **0.919(0.277,1.562)** | **0.005** |
|  | Males* | 0.829(0.665,1.034) | 0.097 |  | **-2.986(-4.481,-1.492)** | **<0.001** |  | **1.362(0.377,2.347)** | **0.007** |
|  | Females* | **0.637(0.507,0.802)** | **<0.001** |  | **-5.852(-7.213,-4.491)** | **<0.001** |  | 0.584(-0.261,1.428) | 0.176 |

adjusted for age, gender (male or female), household income (<12 000 yuan, 12 000-19 999 yuan, 20 000-59 999 yuan, 60 000-99 999 yuan, ≥ 100 000 yuan), family history of hypertension (yes or no), smoking status (never, former, and current), alcohol drinking (continuous), physical activity (continuous), total energy intake per day (continuous), DASH score (continuous), snoring (no, occasionally, habitual), BMI (continuous), waist circumference (continuous), dyslipidemia (yes or no), diabetes (yes or no)

*without adjustment for gender
